# Mitochondrial Mistranslation in Brain Provokes a Metabolic Response Which Mitigates the Age-Associated Decline in Mitochondrial Gene Expression

**DOI:** 10.3390/ijms22052746

**Published:** 2021-03-09

**Authors:** Dimitri Shcherbakov, Reda Juskeviciene, Adrián Cortés Sanchón, Margarita Brilkova, Hubert Rehrauer, Endre Laczko, Erik C. Böttger

**Affiliations:** 1 Institut für Medizinische Mikrobiologie, Universität Zürich, 8006 Zürich, Switzerland; dscherbakov@imm.uzh.ch (D.S.); reda.juskeviciene@gmx.ch (R.J.); acortes@imm.uzh.ch (A.C.S.); mbrilkova@imm.uzh.ch (M.B.); 2 Functional Genomics Center Zurich, ETH Zürich und Universität Zürich, 8006 Zürich, Switzerland; hubert.rehrauer@fgcz.ethz.ch (H.R.); endre.laczko@fgcz.uzh.ch (E.L.)

**Keywords:** mitochondria, misreading, brain, aging, metabolome

## Abstract

Mitochondrial misreading, conferred by mutation V338Y in mitoribosomal protein Mrps5, in-vivo is associated with a subtle neurological phenotype. Brain mitochondria of homozygous knock-in mutant Mrps5^V338Y/V338Y^ mice show decreased oxygen consumption and reduced ATP levels. Using a combination of unbiased RNA-Seq with untargeted metabolomics, we here demonstrate a concerted response, which alleviates the impaired functionality of OXPHOS complexes in Mrps5 mutant mice. This concerted response mitigates the age-associated decline in mitochondrial gene expression and compensates for impaired respiration by transcriptional upregulation of OXPHOS components together with anaplerotic replenishment of the TCA cycle (pyruvate, 2-ketoglutarate).

## 1. Introduction

Mitochondria are multi-functional organelles involved in oxidative metabolism, cellular homeostasis, and signal transduction [1,2,3]. As the site of oxidative phosphorylation, mitochondria convert the energy from nutrients into ATP. To perform their key role in cellular energy production, mitochondria use intricate systems that encompass the breakdown of glucose and fatty acids, coupled to oxidative phosphorylation via the citrate cycle (TCA) [4,5]. Mitochondrial diseases are clinically diverse and may manifest in a tissue-specific or multisystemic manner. Most often they affect postmitotic tissues with high energy demands such as brain, skeletal muscle, heart, or cochlea [6,7,8].

The vast majority of proteins essential for mitochondrial function are nuclear encoded; only 13 of the more than 100 different proteins that make up the mitochondrial oxidative system are encoded by mitochondrial DNA and translated by the mitoribosome [9,10]. The amino acid (aa) replacement V338Y in mouse mitoribosomal protein Mrps5 (uS5) is a recently identified ribosomal ambiguity mutation (*ram*), which decreases translational accuracy and confers mitoribosomal misreading by increasing the incorporation of near-cognate aa-tRNA species [11]. Despite the universal presence of mitochondria, homozygous knock-in C57/BL6 mice carrying the Mrps5 V338Y mutation display a remarkable absence of overt pathology in vivo. The phenotype presented by mutant *MRPS5^V338Y/V338Y^* mice is subtle and mainly composed of neurological stress intolerance and anxiety-related behavioral alterations [11]. Brain mitochondria from *MRPS5^V338Y/V338Y^* mice show impaired mitochondrial function, as revealed by a decline in total oxygen consumption rate (OCR), reduced levels of ATP, and increased amounts of mitochondrial reactive oxygen species (mtROS) [11].

To further assess the alterations induced by mitochondrial misreading in brain of *MRPS5^V338Y/V338Y^* mutant mice at the molecular level, we here used unbiased whole genome RNA-Seq combined with metabolome analysis. For profiling, wild-type and V338Y mutant female mice were sampled at three and 19 months of age for investigations at transcriptome level, together with metabolome studies at 19 months of age.

## 2. Results

### 2.1. Transcriptome Sequencing and Age-Related Changes

Transcriptomes of total cortex tissue samples from wild-type and *MRPS5^V338Y/V338Y^* mutant mice were resolved using RNA-Seq. A total of 16 samples (four for each time-point and genotype) were sequenced. Differential gene expression analysis showed significant transcriptomic changes between young (three months) and old (19 months) animal brain samples. With a threshold *p*-value < 0.05, there were 10,914 and 9355 age-regulated transcripts in wild-type and mutant V338Y datasets, respectively (Supplementary Table S1). Differentially expressed genes (DEGs) with a *p*-value < 1 × 10^−5^ (FDR < 7.31 × 10^−5^), subjected to gene enrichment analysis using online tools EnrichR (https://maayanlab.cloud/Enrichr/ accessed on 8 June 2020) [12] and MetaCore (https://portal.genego.com/ accessed on 8 June 2020), revealed the characteristic age-related changes in both wild-type and V338Y mutant animals [13,14].

Thus, many cellular metabolic processes declined with aging in our mouse cohorts: most prominently mitochondria-related energy production [15,16] (KEGG: oxidative phosphorylation, thermogenesis, citrate cycle, pyruvate metabolism; Wiki: electron transport chain, glycolysis), but also RNA metabolism (KEGG: RNA transport; Wiki: mRNA processing; PN: transcription/transcription by RNA polymerase II), translation (Wiki: translation factors), protein degradation (KEGG: proteasome, ubiquitin-mediated proteolysis; GO: protein polyubiquitination), and synapse-related functions (KEGG: synaptic vesicle cycle, dopaminergic synapse, cholinergic synapse, GABAergic synapse; PN: neurophysiological process/transmission of nerve impulse, development/neurogenesis/synaptogenesis). Upregulated pathways in the aging brain of our mouse cohorts were mostly connected to extracellular matrix reorganization (KEGG: ECM-receptor interaction, cell adhesion molecules; Wiki: focal adhesion; GO: extracellular matrix organization; PN: cell adhesion/cell-matrix interactions) and inflammation (KEGG: complement and coagulation cascade; Wiki: inflammatory response pathway; GO: neutrophil mediated immunity) (Supplementary Figure S1).

### 2.2. Mutation-Related Transcriptome Changes in 19-Month-Old Animals

**Figure 1:**
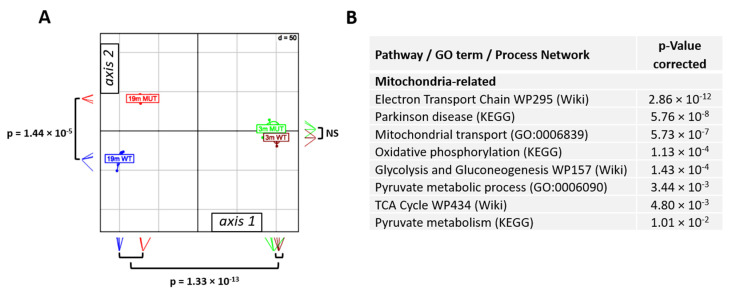
Brain transcriptome analysis. (**A**) Between group analysis (BGA) scatter plot for brain transcriptome profiles (*n* = 4 for three months old *MRPS5 WT*—brown; *n* = 4 for three-month-old *MRPS5 V338Y*—green; *n* = 4 for 19-month-old *MRPS5 WT*—blue; *n* = 4 for 19-month-old *MRPS5 V338Y*—red; NS—not significant). (**B**) Gene enrichment analysis comparing 19-month old *MRPS5 V338Y* and *MRPS5 WT* mice, terms and significance for selected upregulated gene transcripts in mutants, adjusted *p*-values are shown.

For a further assessment of differences, we performed a between group analysis (BGA). BGA simplifies a dataset by reducing its dimensionality and defining axes that best discriminate the groups. We plotted the four groups (three-month-old wild-type, three-month-old V338Y, 19-month-old wild-type, 19-month-old V338Y) into two-dimensional space using the first two most important axes. BGA showed a similar pattern for wild-type and V338Y mutant animals at three months of age. At 19 months of age, the transcriptomic profiles of both genotypes were significantly different from the three-month-old animals. In addition, the 19-month-old V338Y mice were distinct from the age-matched controls, indicating that both age and genotype contribute to the transcriptomic changes. As the three-month- and 19-month-old animals were separated along axis 1 of the scatter plot (Figure 1A), we conclude that axis 1 captures the largest fraction of age-related variance, and thus represents aging, while axis 2 most likely captures the changes associated with the second variable in this group of animals, the genotype, and thus represents the mutation effect (*p*-value 19-month-old V338Y/19 months wt: 1.4 × 10^−5^).

Direct comparison of 19-month-old wild-type and V338Y mutant animals revealed 1761 differentially regulated genes at *p*-value < 0.01 (FDR < 0.09) (see Supplementary Table S1C). Subjecting those DEGs to pathway analysis revealed that the most prominent terms upregulated in V338Y mutants relative to wild-type were mitochondria-related, i.e., oxidative phosphorylation, electron transport chain, mitochondrial transport, pyruvate metabolism, citrate cycle, and glycolysis (see Figure 1).

**Figure 2:**
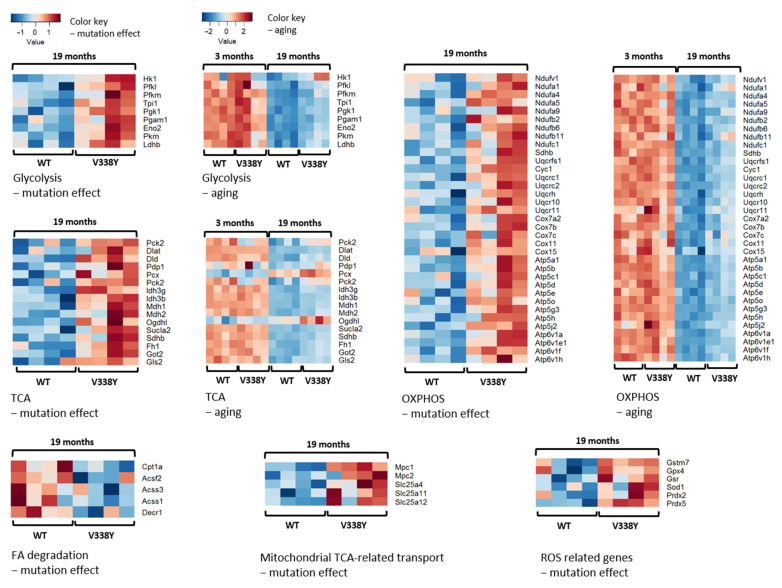
Brain transcriptome analysis. Heatmaps of the genes involved in the mitochondria-associated pathways, regulated in brain of 19-month-old *MRPS5 V338Y* mutant mice, *p*-value < 0.05. Please note the different scales for aging and mutation effect.

Compared to 19-month-old wild-type mice, a large number of OXPHOS-related genes showed significantly increased transcript levels in the 19-month-old V338Y mutants, as did key enzymes of the citrate cycle (see heatmaps Figure 2 and Table 1 for individual genes), e.g., isocitrate dehydrogenase complex subunits *Idh3b* and *Idh3g*, which catalyze irreversible rate-limiting steps, malate dehydrogenase (*Mdh2*), oxoglutarate dehydrogenase (*Ogdh1*, *Dld*), succinate-CoA ligase (*Sucla2*), succinate dehydrogenase (*Sdhb*), and fumarate hydratase (*Fh1*).

**Figure 3:**
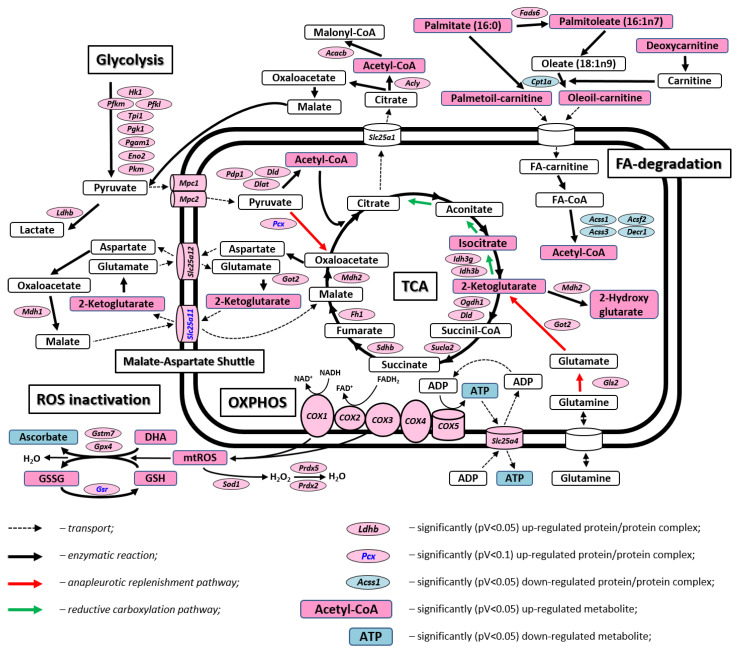
Scheme of the mitochondria-associated metabolic pathways affected in mouse brain by mutation *MRPS5 V338Y*.

Transcriptional activation of the citrate cycle came along with significantly increased transcript levels for key enzymes of glycolysis, e.g., hexokinase 1 (*Hk1*), phosphofructokinases (*Pfk1*, *Pfkm*), phosphoglycerate kinase 1 (*Pgk1*), and pyruvate kinase (*Pkm*). Increased transcript levels for the mitochondrial pyruvate carrier MPC1 and MPC2 indicate that the excess of pyruvate derived from glycolysis is transported into the mitochondria, where it enters the TCA either as acetyl-CoA (*Pdp1*, *Dlat*, *Dld*) or as anaplerotic replenishment by means of pyruvate carboxylase (*Pcx*), which catalyzes the transformation of pyruvate to oxaloacetate. Additional anaplerotic replenishment of the TCA by α-ketoglutarate is indicated by increased transcript levels of *Gls2* and *Got2* (see Figure 3).

In contrast to the increased flow through TCA and ETC, we find significant depletion of genes involved in mitochondrial transport and beta-oxidation of fatty acids, i.e., cytosolic carnitine palmitoyltranferase (*Cpt1a*), mitochondrial acyl-synthetases (*Acsf2*, *Accs1*, *Acss3*), and 2,4 dienoyl-CoA reductase (*Decr1*), the latter participating in the beta-oxidation of unsaturated FAs.

Finally, we find increased transcript levels for several ROS-protective enzymes, e.g., superoxide dismutase (*Sod1*), peroxiredoxins (*Prdx2*, *Prdx5*), glutathione-S-transferase (*Gstm7*), and glutathione peroxidase (*Gpx4*).

Selected target genes (OXPHOS complex I and complex V components *Cox11* and *Atp5s*, mitochondrial pyruvate transporter *Mpc1*, ROS-protector peroxiredoxine *Prdx5*) were chosen to validate the RNA-Seq data by RT-qPCR. The differences in gene expression levels determined by RT-qPCR were consistent with the transcriptome data (Supplementary Figure S2).

### 2.3. Mutation-Related Metabolic Changes in 19-Month-Old Animals

To investigate whether in addition to the transcriptome changes, the mutation V338Y manifests at the level of metabolic alterations, we performed untargeted metabolomic profiling in brain cerebellum samples of 19-month-old wild-type and V338Y mutants; 304 metabolites common to all wild-type and mutant samples were identified. After removing redundant and false positives, 50 metabolites were found to be significantly altered in V338Y mutants compared to wild-type mice. Most importantly, we found that TCA intermediates acetyl-CoA, isocitrate, and α-ketoglutarate were significantly enriched in the V338Y mutants (Table 2). Increased levels of TCA intermediates came together with an accumulation of intermediates of fatty acid catabolism: oleoylcarnitine, palmitoylcarnitine, acetylcarnitine, and the carnitine precursor deoxycarnitine. In addition, we find elevated levels of both forms of glutathione in the V338Y mutant—oxidized (GSSG) and reduced (GSH) glutathione, with a resulting relative increase in the GSSG/GSH ratio (ratio GSSG/GSH was 0.51 for wild-type and 0.70 for mutant animals).

## 3. Discussion

We have previously shown that brain mitochondria of mice carrying the homozygous knock-in mutation *MRPS5 V338Y* show impaired mitochondrial function with decreased oxygen consumption, reduced ATP content, and increased levels of reactive oxygen species [11]. Presumably, impaired mitochondrial function in these mice is the result of less functional mitochondrial OXOHOS proteins synthesized on mistranslating V338Y mitoribosomes. We here used whole genome transcriptome analysis together with metabolic studies to further profile the molecular alterations associated with mitochondrial misreading. Our data indicate a concerted transcriptional and metabolic response to mitigate impaired functionality of OXPHOS complexes (see Figure 3). This response includes (i) elevated gene transcripts of nuclear-encoded components of OXPHOS complexes; (ii) transcriptional upregulation of glycolysis and TCA providing NADH and FADH_2_ to fuel oxidative phosphorylation; and (iii) increased transcript levels of ROS-protective enzymes. As a result of increased glycolysis, elevated levels of pyruvate and NADH can either be converted to lactate and NAD^+^ by lactate dehydrogenase or transported into mitochondria for further utilization in the TCA. Together with transcriptional upregulation of glycolytic enzymes, mutant V338Y mice show increased transcript levels of both lactate dehydrogenase (*Ldhb*) and pyruvate transporters MPC1/MPC2 combined with components of the malate-aspartate shuttle involved in NADH transport (*Mdh1*, *Got2*, *Slc25a12*). Inside mitochondria, pyruvate can enter the TCA either as oxaloacetate (increased levels of pyruvate carboxylase *Pcx*) or as acetyl-CoA (increased levels of pyruvate-dehydrogenase complex *Dlat* and *Dld* and positive regulator *Pdp1*). Transcriptional upregulation of *Gls2* and *Got2* points to additional anaplerotic replenishment of the TCA cycle by 2-ketoglutarate. Together with more active glycolysis, pyruvate/NADH transport, and anaplerotic replenishment, transcriptional upregulation of the TCA cycle leads to enhanced mitochondrial NADH levels. However, the functional impairment of oxidative phosphorylation in the brain of *MRPS5 V338Y* mutant mice, as indicated by reduced levels of total oxygen consumption, would hinder the effective oxidation of extra NADH, thus slowing down TCA reactions and leading to an accumulation of TCA intermediates (isocitrate, 2-ketoglutarate) and substrate acetyl-CoA. Supporting this finding, we note increased levels of the TCA side-product 2-hydroxyglutarate (fold change 1.71, *p*-value 0.123), which is a marker of mitochondrial reductive stress caused by respiratory chain dysfunction [17]. In contrast to isocitrate and α-ketoglutarate, there is no significant difference for citrate in V338Y mutants compared to wild-type (fold change 0.99, *p*-value 0.85). Interconversion of citrate and isocitrate is necessary for the TCA cycle to operate in either direction. Changes in this equilibrium with elevated levels of isocitrate or perturbations that result in an increase in the α-ketoglutarate/citrate ratio, as both observed in the V338Y mutants, would favor reductive metabolism [18]. Reductive flux via the TCA cycle culminates in the export of mitochondrial citrate into the cytosol and conversion to cytosolic acetyl-CoA and further to malonyl-CoA via ATP citrate lyase and acetyl-CoA carboxylase (increased levels for *Acly* and *Acacb* are found in the V338Y mutants).

We observed significant changes in brain fatty acid (FA) metabolism of *MRPS5 V338Y* mutant mice both at the transcriptomic and metabolomic level. While the brain’s capacity for FA oxidation is limited [19], astrocytes can in part use FA oxidation to support their energetic demands [20]. In brain of *MRPS5* mutant mice, accumulation of FA-catabolic intermediates oleoylcarnitine, palmitoylcarnitine, butyrylcarnitine (*p*-value 0.118), acetylcarnitine, and carnitine precursor deoxycarnitine comes together with decreased transcript levels of genes and enzymes responsible for transport of FAs into mitochondria and subsequent β-oxidation (*Cpt1a*, *Acsf2*, *Acss1*, *Acss3*, *Decr1*). Inhibition of FA-oxidation has been observed as a result of impaired oxidative phosphorylation [21]. Presumably, under conditions of less effective OXPHOS, glycolysis is favored over FA-oxidation for ATP generation, as compared to glycolysis FA-oxidation requires higher oxygen consumption.

With interest, we note that skeletal muscle and brain, sampled in parallel from the identical cohorts of *MRPS5^V338Y/V338Y^* mutant mice, despite sharing the characteristic of postmitotic tissue, show disparate responses to impaired function of the 13 mtDNA encoded OXPHOS proteins. While for both tissues the effect of the *MRPS5 V338Y* mutation is age-dependent, we find bypassing of ETC by increased glycolysis, pentose phosphate pathway, fatty acid synthesis, and generation of lipid droplets in muscle [22], compared to increased flow through TCA and ETC in brain. Presumably, the latter reflects the dependency of neuronal cells on energy production via glycolysis feeding into TCA and ATP generation by oxidative phosphorylation. Dichotomic responses to disruption of oxidative phosphorylation have also been observed in *C. elegans* with compensation either by increasing ETC activity and feeding more substrates into the TCA cycle, or by bypassing ETC and upregulation of glycolysis, glyoxylate cycle, and glycerol fermentation [23].

Most prominent in aging is the decline of mitochondria-related energy production [15,16]. The significant upregulation of transcripts related to mitochondrial metabolism in V338Y mutant mice, e.g., oxidative phosphorylation, glycolysis, and TCA cycle, reflects a compensatory response, which even at the level of individual pathway-specific genes mitigates their age-related decline (Supplementary Table S2). We note that this comes along with less pronounced enrichment of age-associated gene expression related to extracellular matrix and inflammation in the V338Y mutants (Supplementary Table S3). We hypothesize that the transcriptomic alterations observed in brain of the 19-month-old V338Y mutant animals are part of a mitohormetic response [24,25,26] to the mildly impaired function of the mtDNA encoded OXPHOS proteins generated by mistranslating mitoribosomes.

## 4. Materials and Methods

### 4.1. Animals

The transgenic *MRPS5^V338Y/V338Y^* mouse strain has been previously described [11]. Animal experiments were approved by the Veterinary Office of the Canton of Zurich (licenses 29/2012 and 44/2015). 

Expression levels of *MRPS5* mRNA were comparable in the brain of mutant and wild-type mice, indicating that the mutation did not lead to deregulation of *MRPS5* gene expression. The animals were sacrificed at three and 19 months of age; the brain was carefully isolated, split to cortex and cerebellum, snap frozen, and stored at −80 °C.

### 4.2. RNA Extraction

The following mice were used for the analysis: four three-month-old female animals of *MRPS5^WT/WT^*, four three-month-old female animals of *MRPS5^V338Y/V338Y^*, four 19-month-old female animals of *MRPS5^WT/WT^*, and four 19-month-old female animals of *MRPS5^V338Y/V338Y^*. Deep frozen cortex was grinded under liquid nitrogen, and RNA was extracted using TRIzol reagent (Invitrogen, Carlsbad, CA, USA) according to the manufacturer’s instructions. The quality of the isolated RNA was assessed using a Tapestation 2200 (Agilent Technologies, Santa Clara, CA, USA). Only those samples with a 260/280 nm ratio between 1.8–2.1 and a 28S/18S ratio within 1.5–2 were further processed; all samples used for RNA sequencing had RIN (RNA Integrity Number) ≥ 7.5.

### 4.3. cDNA Library Preparation and Sequencing

RNA sequencing (RNA-Seq) was performed at the UZH/ETH Functional Genomics Center Zurich (FGCZ) according to the Illumina RNA sequencing protocol. The TruSeq Stranded mRNA Sample Prep Kit (Illumina, San Diego, CA, USA) was used in the succeeding steps. Briefly, total RNA samples (100–1000 ng) were ribosome depleted and then reverse-transcribed into double-stranded cDNA with actinomycin added during first-strand synthesis. The cDNA samples were fragmented, end-repaired, and polyadenylated. TruSeq adapters containing the index for multiplexing were ligated to the fragmented DNA samples. Fragments containing TruSeq adapters on both ends were selectively enriched with PCR. The quality and quantity of the enriched libraries were validated using Qubit^®^ (1.0) Fluorometer and the Caliper GX LabChip^®^ GX (Caliper Life Sciences, Hopkinton, MA, USA). The product was a smear with an average fragment size of approximately 360 bp. The libraries were normalized to 10 nM in Tris-Cl 10 mM, pH 8.5, with 0.1% Tween 20. The TruSeq SR Cluster Kit v4-cBot-HS (Illumina, San Diego, CA, USA) was used for cluster generation using 8 pM of pooled normalized libraries on the cBOT. Sequencing was performed on the Illumina HiSeq 2500 single end 126 bp using the TruSeq SBS Kit v4-HS (Illumina, San Diego, CA, USA).

### 4.4. Transcriptome Data Analysis

The quality of the reads was assessed using FastQC (Babraham Bioinformatics (http://www.bioinformatics.babraham.ac.uk/projects/fastqc/ accessed on 8 January 2017), and potential contaminations were evaluated with FastQ Screen (Babraham Bioinformatics (http://www.bioinformatics.babraham.ac.uk/projects/fastq_screen/ accessed on 8 January 2017) using bowtie2 vs. 2.1.0 [27] default parameters. Quantification of gene expression was performed using the RSEM package (version 1.2.18) [28] mapping against the Ensembl 75 annotations derived from the mouse genome assembly GRCm37. Genes not present (<10 counts per gene) in at least 50% of samples from one condition were discarded from further analyses. Differential gene expression analysis between sample groups of interest was performed using the R/bioconductor package edgeR [29]. Differences in gene expression levels were calculated as log2 fold changes; resulting *p*-values were adjusted using Benjamini–Hochberg multiple test correction [30]. An adjusted *p*-value < 0.05 (for comparison 19-month-old WT vs. V338Y mutant samples) and <0.00001 (for comparison 19-month-old vs. three-month-old WT or V338Y mutant samples) was considered statistically significant; no fold change threshold was applied.

BGA analysis was done using R/bioconductor package MADE4 [31]. Genes used for this analysis included all genes with adjusted *p*-values < 0.05 (for comparison 19-month-old WT vs. three-month-old WT and 19-month-old V338Y mutant vs. three-month-old V338Y mutant).

Functional annotation of differentially expressed genes and pathway enrichment analysis was performed using the online biological information tool EnrichR (http://amp.pharm.mssm.edu/Enrichr/ accessed on 8 June 2020) [12] that integrates several biological databases and provides a comprehensive set of functional annotation information on genes and proteins. The databases KEGG (Kyoto Encyclopedia of Genes and Genomes, http://www.genome.jp/kegg/pathway.html accessed on 8 June 2020) [32], WP (WikiPathways, https://www.wikipathways.org accessed on 8 June 2020) [33], and GO (Gene Ontology enrichment, http://www.geneontology.org accessed on 8 June 2020) [34] from EnrichR were used for analysis. In addition, Process Networks analysis was done using the online tool MetaCore (Clarivate Analytics, Philadelphia, PA, USA, https://portal.genego.com/ accessed on 8 June 2020). The Benjamini–Hochberg false discovery rate (FDR) procedure was applied to pathway analysis as a correction for multiple testing. Pathways or terms with FDR corrected *p*-value (adjusted *p*-value) < 0.05 were considered statistically significant.

### 4.5. qRT-PCR

To confirm the results of transcriptome analysis, selected genes were subjected to qRT-PCR (for list of genes and primers see Supplementary Table S4). RNA samples were reverse transcribed into cDNA using High Capacity RNA-to-DNA Kit (Applied Biosystems, Foster City, CA, USA). cDNA was analyzed by real-time quantitative PCR (qPCR) using an ABI 7500 Fast Real Time PCR system (Applied Biosystems, Foster City, CA, USA) and a pair of gene-specific primers for each selected gene. qPCR was performed in triplicates using EvaGreen Mix (Bio&SELL, Feucht, Germany) and 20 ng of cDNA per reaction. The transcript levels were normalized to the geometric mean of three housekeeping genes (*Hmbs*, *Ywhaz*, *Actb*) [35,36] from the same sample used as an internal reference, and the fold change of mutant relative to the WT mice was calculated as 2^−ΔΔCT^ [37]. Statistical analysis was performed with GraphPad Prism 5.0 software; unpaired Student’s *t*-test was used to estimate significance.

### 4.6. Metabolome Analysis

Cerebellums isolated from ten 19-month-old female animals of *MRPS5^WT/WT^* and five 19-month-old female animals of *MRPS5^V338Y/V338Y^* were used for metabolome analysis. Metabolome analysis was performed by Metabolon (Morrisville, NC, USA). In brief, samples were prepared by proprietary series of organic and aqueous extractions in order to remove proteins and to recover maximum amount of small molecules. The extracted samples were split in equal parts and analyzed via GC-MS or LC-MS/MS. For the LC-MS/MS two equal parts were analyzed in the positive (acidic solvent) and in the negative (basic solvent) ionization mode. Samples for GCMS were bistrimethyl-silyl-trifluoroacetamide derivatized and were run with a 5% diphenyl/95% dimethyl polysiloxane fused silica column. Obtained data were combined and normalized. Welch’s *t*-test with Holm–Bonferroni correction was used for statistical analysis.

### 4.7. Database Submission

Transcriptome data are available in Gene Expression Omnibus (GEO), accession number GSE121395, token mbitkoqatfufdkn.

Complete original metabolome dataset is included in Supplementary Data (Supplementary Table S5).

## Figures and Tables

**Table 1:** Regulated genes related to mitochondria-associated metabolism in 19-month-old *MRPS5^V338Y/V338Y^* mutant compared to wild-type mice.

Gene Name		Fold Change	*p*-Value
Glycolysis			
hexokinase 1	*Hk1*	1.07	2.09 × 10^−2^
hexokinase 3	*Hk3*	0.79	2.41 × 10^−2^
phosphofructokinase, liver, B-type	*Pfkl*	1.09	5.27 × 10^−3^
phosphofructokinase, muscle	*Pfkm*	1.08	1.22 × 10^−2^
triosephosphate isomerase 1	*Tpi1*	1.09	6.05 × 10^−3^
phosphoglycerate kinase 1	*Pgk1*	1.08	1.24 × 10^−2^
phosphoglycerate mutase 1	*Pgam1*	1.10	9.78 × 10^−3^
enolase 2	*Eno2*	1.12	2.63 × 10^−4^
pyruvate kinase, muscle	*Pkm*	1.10	3.32 × 10^−3^
lactate dehydrogenase B	*Ldhb*	1.07	2.75 × 10^−2^
**Mitochondrial transporters**			
mitochondrial pyruvate carrier 1	*Mpc1*	1.19	2.42 × 10^−5^
mitochondrial pyruvate carrier 2	*Mpc2*	1.08	3.99 × 10^−2^
solute carrier family 25 member 4 (mitochondrial ATP/ADP translocator)	*Slc25a4*	1.10	2.70 × 10^−3^
solute carrier family 25 member 12 (mitochondrial aspartate/glutamate antiporter Aralar)	*Slc25a12*	1.07	2.54 × 10^−2^
**TCA and related processes**			
dihydrolipoamide S-acetyltransferase (component of pyruvate dehydrogenase complex)	*Dlat*	1.07	4.00 × 10^−2^
dihydrolipoamide dehydrogenase	*Dld*	1.16	1.17 × 10^−5^
pyruvate dehyrogenase phosphatase catalytic subunit 1	*Pdp1*	1.08	1.73 × 10^−2^
isocitrate dehydrogenase 3 (NAD+), gamma	*Idh3g*	1.10	4.79 × 10^−3^
isocitrate dehydrogenase 3 (NAD+) beta	*Idh3b*	1.07	2.53 × 10^−2^
malate dehydrogenase 1, NAD (cytosolic)	*Mdh1*	1.13	1.42 × 10^−4^
malate dehydrogenase 2, NAD (mitochondrial)	*Mdh2*	1.08	8.42 × 10^−3^
oxoglutarate dehydrogenase-like (mitochondrial)	*Ogdhl*	1.10	1.56 × 10^−3^
succinate-Coenzyme A ligase, ADP-forming, beta subunit	*Sucla2*	1.07	2.56 × 10^−2^
succinate dehydrogenase complex, subunit B	*Sdhb*	1.07	3.86 × 10^−2^
fumarate hydratase 1	*Fh1*	1.08	3.16 × 10^−2^
glutamate oxaloacetate transaminase 2 (mitochondrial)	*Got2*	1.13	1.05 × 10^−4^
ATP citrate lyase	*Acly*	1.09	4.38 × 10^−3^
acetyl-Coenzyme A carboxylase beta	*Acacb*	1.23	4.84 × 10^−5^
**Fatty acid transport and degradation**			
carnitine palmitoyltransferase 1a, liver	*Cpt1a*	0.90	2.77 × 10^−3^
acyl-CoA synthetase family member 2	*Acsf2*	0.80	1.61 × 10^−3^
acyl-CoA synthetase short-chain family member 3	*Acss3*	0.82	3.30 × 10^−2^
acyl-CoA synthetase short-chain family member 1	*Acss1*	0.91	4.41 × 10^−2^
2,4-dienoyl CoA reductase 1, mitochondrial	*Decr1*	0.89	1.20 × 10^−2^
**Glutathione metabolism and ROS**			
glutathione S-transferase, mu7	*Gstm7*	1.15	2.43 × 10^−2^
glutathione peroxidase 4	*Gpx4*	1.07	3.82 × 10^−2^
superoxide dismutase 1, cytosolic	*Sod1*	1.07	3.84 × 10^−2^
peroxiredoxin 5	*Prdx5*	1.17	1.32 × 10^−6^
peroxiredoxin 2	*Prdx2*	1.09	5.83 × 10^−3^

Only genes with a *p*-value ≤ 0.05 are shown.

**Table 2:** Selected metabolites with different pool sizes in 19-month-old *MRPS5^V338Y/V338Y^* mutant compared to wild-type mice.

Metabolite	Fold Change (19M *MRPS5^V338Y/V338Y^* vs. *MRPS5^WT/WT^*)	*p*-Value (Welch’s t-Test)
TCA		
alpha-ketoglutarate	1.19	0.003
isocitrate	1.69	0.004
acetyl CoA	1.38	0.014
**FA degradation**		
deoxycarnitine	1.21	0.096
acetylcarnitine	1.38	0.028
oleoylcarnitine	1.80	0.056
palmitoylcarnitine	1.60	0.098
**Ascorbate metabolism**		
dehydroascorbate	1.21	0.050
ascorbate (Vitamin C)	0.44	0.062
**Glutathione metabolism**		
glutathione, oxidized (GSSG)	1.92	0.003
glutathione, reduced (GSH)	1.39	0.048

Only metabolites with a *p*-value < 0.1 are shown.

## Data Availability

Transcriptome data are available in Gene Expression Omnibus (GEO), accession number GSE121395. Complete original metabolome dataset is included in Supplementary Data (Table S5).

## Supplementary Materials

The following are available online at https://www.mdpi.com/1422-0067/22/5/2746/s1, Figure S1: Brain RNAseq analysis of *Mrps5^WT/WT^* and *Mrps5^V338Y/V338Y^* mice of 3 months and 19 months age. Figure S2: RT-qPCR for selected genes in brain of *MRPS5^V338Y/V338Y^* and *MRPS5^WT/WT^* mice of 19 months age. Table S1: RNA-Seq analysis of *MRPS5^WT/WT^* and *MRPS5^V338Y/V338Y^* brain from mice 3 months and 19 months of age. Table S2: Individual genes related to mitochondria-associated metabolism, differently expressed in brain of 19 months old *MRPS5^WT/WT^* mice in comparison to 3 months old *MRPS5^WT/WT^* mice. Table S3: Gene enrichment analysis comparing 19 months old *MRPS5^WT/WT^* and *MRPS5^V338Y/V338Y^* mice. Table S4: Primers used for real-time quantitative PCR of target and house-keeping genes. Table S5: Metabolomic analysis of *MRPS5^WT/WT^* and *MRPS5^V338Y/V338Y^* cerebellum from mice 19 months of age.
