# Supplementary material for: Mitochondrial Mistranslation in Brain Provokes a Metabolic Response Which Mitigates the Age-Associated Decline in Mitochondrial Gene Expression
Source: Int J Mol Sci. 2021 Mar 9;22(5):2746. doi: 10.3390/ijms22052746 (PMC7963198; doi:10.3390/ijms22052746)
Supplement: Supplementary file 1 [file ijms-22-02746-s001.zip › Supplementary Figures S1-S2.pdf]

Pathway/GO term/Process Network	p-value corrected
**Downregulated terms**	
**Mitochondria-related**	
Oxidative phosphorylation (KEGG)	8.61E-25
Electron Transport Chain WP295 (Wiki)	2.13E-19
Thermogenesis (KEGG)	1.51E-17
Mitochondrial respiratory chain complex assembly (GO)	6.18E-08
Citrate cycle (KEGG)	4.45E-07
Glycolysis and Gluconeogenesis WP157 (Wiki)	2.21E-04
Pyruvate metabolism (KEGG)	3.90E-03
**Translation**	
Translation Factors WP307 (Wiki)	1.14E-02
**RNA metabolism**	
RNA transport (KEGG)	2.24E-06
Transcription. Transcription by RNA polymerase II (PN)	2.19E-03
mRNA processing WP310 (Wiki)	9.81E-02
**Proteasome-related**	
Proteasome (KEGG)	1.45E-14
Ubiquitin mediated proteolysis (KEGG)	1.67E-08
Protein polyubiquitination (GO)	4.77E-05
**Synapse-related**	
Synaptic vesicle cycle (KEGG)	9.40E-06
Dopaminergic synapse (KEGG)	1.56E-04
Neurophysiological process. Transmission of nerve impulse (PN)	2.91E-04
Development. Neurogenesis. Synaptogenesis (PN)	6.04E-04
Cholinergic synapse (KEGG)	3.37E-02
GABAergic synapse (KEGG)	4.69E-02
**Upregulated terms**	
**Extracellular matrix reorganization/tissue remodeling**	
Extracellular matrix organization (GO)	8.06E-33
Cell adhesion. Cell-matrix interactions (PN)	3.60E-25
Focal Adhesion WP85 (Wiki)	4.96E-17
ECM-receptor interaction (KEGG)	1.62E-16
Cell adhesion molecules (KEGG)	4.40E-12
**Inflammation**	
Neutrophil mediated immunity (GO)	5.42E-13
Inflammatory Response Pathway WP458 (Wiki)	3.80E-06
Complement and coagulation cascades (KEGG)	7.14E-06

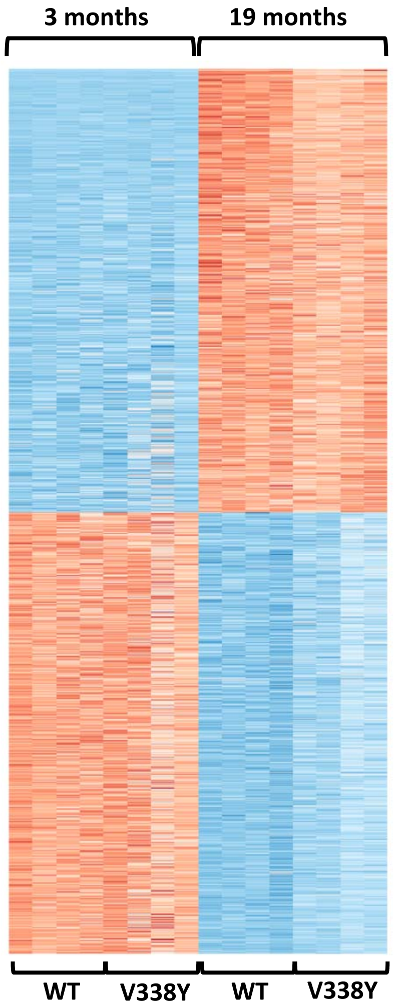

Figure S1. Brain RNAseq analysis of Mrps5$^{WT/WT}$ and Mrps5$^{V338Y/V338Y}$ mice of 3 months and 19 months age. (Left): Terms and significance for regulated transcripts (19 months *vs* 3 months), adjusted p-values are shown. (Right) Heatmap of genes, significantly regulated during aging ($p < 1\times10^{-5}$).

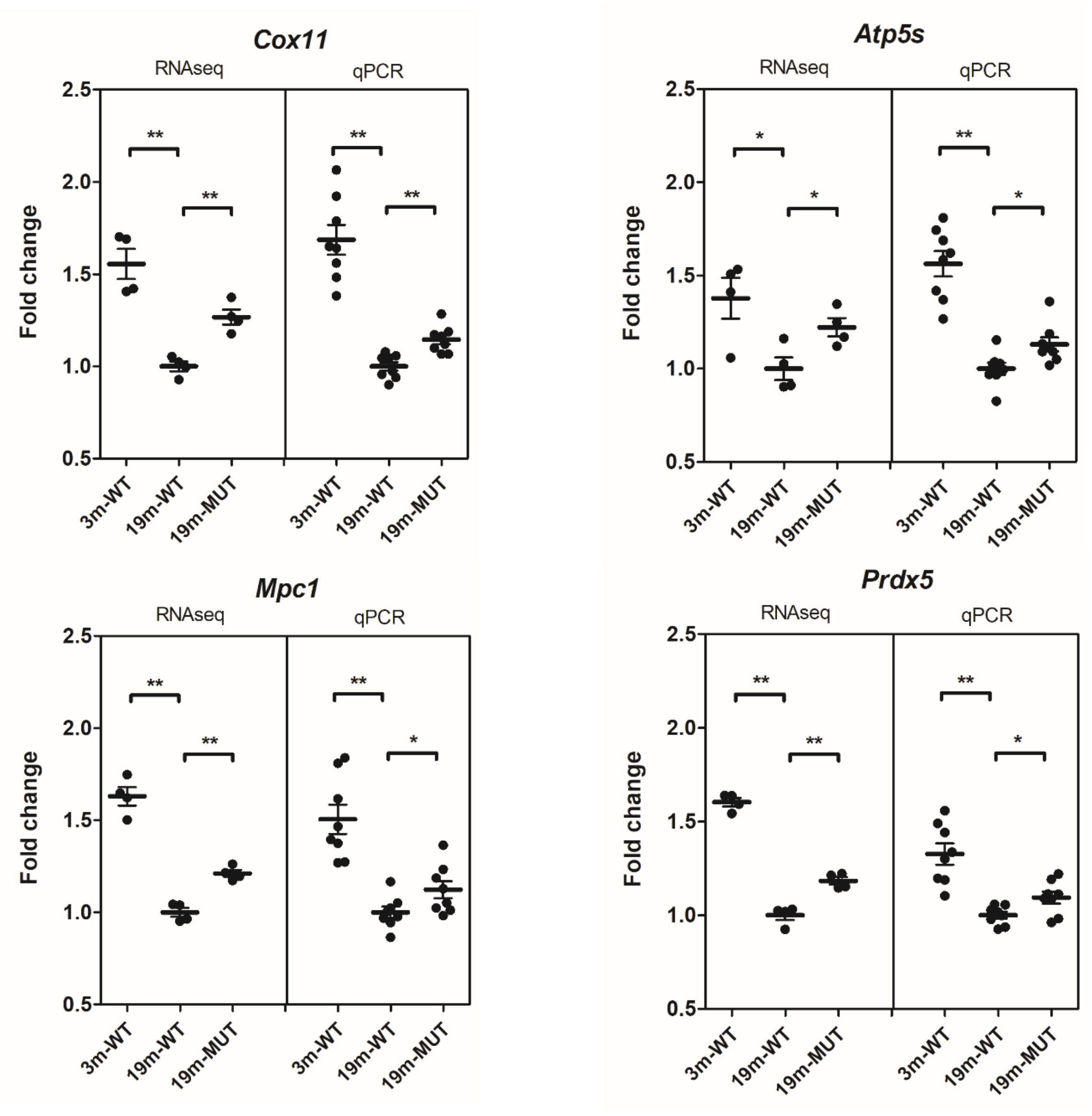

Figure S2. RT-qPCR for selected genes in brain of Mrps5$^{V338Y/V338Y}$ and Mrps5$^{WT/WT}$ mice of 19 months age; 3 months Mrps5$^{WT/WT}$ and RNAseq data are shown for comparison. *- $p < 0.05$, **- $p < 0.001$. For RNAseq biological samples (n=4) are shown. For qPCR technical duplicates for biological samples (n=4) are shown. Error bars represent SEM.
