# Supplementary material for: Mitochondrial Mistranslation in Brain Provokes a Metabolic Response Which Mitigates the Age-Associated Decline in Mitochondrial Gene Expression
Source: Int J Mol Sci. 2021 Mar 9;22(5):2746. doi: 10.3390/ijms22052746 (PMC7963198; doi:10.3390/ijms22052746)
Supplement: Supplementary file 1 [file ijms-22-02746-s001.zip › Supplementary Tables S1-S4.docx]

**Supplementary Table S1.** RNA-Seq analysis of Mrps5^WT/WT^ and Mrps5^V338Y/V338Y^ brain from mice 3 months and 19 months of age. Number of regulated genes at different levels of significance are shown (number of total analyzed features: 39179).

1. **19m WT *vs* 3m WT**

Number of the genes with counts above threshold: 15865.

| **p-Value** | **Significantly**  **regulated** | **FDR** | **Fold change** | | | | |
| --- | --- | --- | --- | --- | --- | --- | --- |
|  |  |  | **FC > 1** | **FC > 1.5** | **FC > 2** | **FC > 3** | **FC > 4** |
| p < 0.1 | 11634 | 0.1362 | 11634 | 3283 | 1169 | 365 | 161 |
| p < 0.05 | 10914 | 0.07263 | 10914 | 3283 | 1169 | 365 | 161 |
| p < 0.01 | 9541 | 0.01662 | 9541 | 3283 | 1169 | 365 | 161 |
| p < 0.001 | 8120 | 0.001948 | 8120 | 3272 | 1169 | 365 | 161 |
| p < 1e-04 | 7026 | 0.0002243 | 7026 | 3099 | 1169 | 365 | 161 |
| p < 1e-05 | 6219 | 0.0000255 | 6219 | 2951 | 1169 | 365 | 161 |

1. **19m MUT *vs* 3m MUT**

Number of the genes with counts above threshold: 16571.

| **p-Value** | **Significantly**  **regulated** | **FDR** | **Fold change** | | | | |
| --- | --- | --- | --- | --- | --- | --- | --- |
|  |  |  | **FC > 1** | **FC > 1.5** | **FC > 2** | **FC > 3** | **FC > 4** |
| p < 0.1 | 10450 | 0.1585 | 10450 | 2719 | 1088 | 311 | 130 |
| p < 0.05 | 9355 | 0.08856 | 9355 | 2708 | 1087 | 311 | 130 |
| p < 0.01 | 7264 | 0.0228 | 7264 | 2669 | 1082 | 311 | 130 |
| p < 0.001 | 5011 | 0.003305 | 5011 | 2363 | 1062 | 310 | 130 |
| p < 1e-04 | 3389 | 0.0004867 | 3389 | 1999 | 1012 | 304 | 126 |
| p < 1e-05 | 2257 | 0.0000731 | 2257 | 1614 | 900 | 285 | 116 |

1. **19m MUT *vs* 19m WT (mutation effect)**

Number of the genes with counts above threshold: 16226.

| **p-Value** | **Significantly**  **regulated** | **FDR** | **Fold change** | | | | |
| --- | --- | --- | --- | --- | --- | --- | --- |
|  |  |  | **FC > 1** | **FC > 1.5** | **FC > 2** | **FC > 3** | **FC > 4** |
| p < 0.1 | 4557 | 0.3558000 | 4557 | 140 | 60 | 30 | 16 |
| p < 0.05 | 3329 | 0.2435000 | 3329 | 140 | 60 | 30 | 16 |
| p < 0.01 | 1761 | 0.0920700 | 1761 | 140 | 60 | 30 | 16 |
| p < 0.001 | 880 | 0.0184300 | 880 | 135 | 60 | 30 | 16 |
| p < 1e-04 | 524 | 0.0030600 | 524 | 123 | 60 | 30 | 16 |
| p < 1e-05 | 347 | 0.0004666 | 347 | 109 | 60 | 30 | 16 |

**Supplementary Table S2.** Individual genes related to mitochondria-associated metabolism, differently expressed in brain of 19 months old Mrps5^WT/WT^ mice in comparison to 3 months old Mrps5^WT/WT^ mice.

| **Gene name** | | **Fold change** | **p-Value** |
| --- | --- | --- | --- |
| **Glycolysis** | |  |  |
| hexokinase 1 | Hk1 | 0.94 | 4.53E-02 |
| hexokinase 3 | Hk3 | 1.80 | 9.90E-07 |
| phosphofructokinase, liver, B-type | Pfkl | 0.79 | 3.29E-12 |
| phosphofructokinase, muscle | Pfkm | 0.77 | 2.13E-17 |
| triosephosphate isomerase 1 | Tpi1 | 0.67 | 2.11E-36 |
| phosphoglycerate kinase 1 | Pgk1 | 0.73 | 1.23E-20 |
| phosphoglycerate mutase 1 | Pgam1 | 0.59 | 3.42E-42 |
| enolase 2 | Eno2 | 0.68 | 5.20E-36 |
| pyruvate kinase, muscle | Pkm | 0.75 | 8.03E-20 |
| lactate dehydrogenase B | Ldhb | 0.81 | 2.72E-11 |
|  | | | |
| **Mitochondrial transporters** | |  |  |
| mitochondrial pyruvate carrier 1 | Mpc1 | 0.63 | 6.03E-27 |
| mitochondrial pyruvate carrier 2 | Mpc2 | 0.68 | 2.68E-20 |
| solute carrier family 25 member 4 (mitochondrial ATP/ADP translocator) | Slc25a4 | 0.67 | 1.84E-37 |
| solute carrier family 25 member 12 (mitochondrial aspartate/glutamate antiporter Aralar) | Slc25a12 | 0.84 | 1.69E-07 |
| solute carrier family 25 member 11 (mitochondrial malate/α-ketoglutarate antiporter) | Slc25a11 | 0.71 | 5.64E-20 |
|  | | | |
| **TCA and related processes** | |  |  |
| dihydrolipoamide S-acetyltransferase (component of pyruvate dehydrogenase complex) | Dlat | 0.57 | 2.48E-59 |
| dihydrolipoamide dehydrogenase | Dld | 0.67 | 1.33E-28 |
| pyruvate dehyrogenase phosphatase catalytic subunit 1 | Pdp1 | 0.91 | 5.04E-03 |
| pyruvate carboxylase | Pcx | 1.14 | 7.30E-05 |
| isocitrate dehydrogenase 3 (NAD+), gamma | Idh3g | 0.73 | 1.05E-19 |
| isocitrate dehydrogenase 3 (NAD+) beta | Idh3b | 0.66 | 1.22E-35 |
| malate dehydrogenase 1, NAD (cytosolic) | Mdh1 | 0.58 | 6.24E-66 |
| malate dehydrogenase 2, NAD (mitochondrial) | Mdh2 | 0.76 | 1.92E-16 |
| oxoglutarate dehydrogenase-like (mitochondrial) | Ogdhl | 1.10 | 4.31E-03 |
| succinate-Coenzyme A ligase, ADP-forming, beta subunit | Sucla2 | 0.63 | 2.20E-42 |
| succinate dehydrogenase complex, subunit B | Sdhb | 0.71 | 2.02E-22 |
| fumarate hydratase 1 | Fh1 | 0.75 | 7.49E-14 |
| glutamate oxaloacetate transaminase 2 (mitochondrial) | Got2 | 1.13 | 2.13E-35 |
|  | | | |
| **Fatty acid transport and degradation** |  |  |  |
| carnitine palmitoyltransferase 1a, liver | Cpt1a | 1.85 | 7.55E-48 |
| acyl-CoA synthetase family member 2 | Acsf2 | 1.86 | 1.69E-14 |
| acyl-CoA synthetase short-chain family member 3 | Acss3 | 2.00 | 7.14E-11 |
| acyl-CoA synthetase short-chain family member 1 | Acss1 | 1.49 | 1.94E-13 |
| 2,4-dienoyl CoA reductase 1, mitochondrial | Decr1 | 1.38 | 1.50E-08 |
|  | | | |
| **Glutathione metabolism and ROS** |  |  |  |
| glutathione S-transferase, mu7 | Gstm7 | 0.81 | 2.45E-03 |
| glutathione peroxidase 4 | Gpx4 | NR | *0.86* |
| glutathione reductase | Gsr | 0.77 | 5.37E-07 |
| superoxide dismutase 1, cytosolic | Sod1 | 0.80 | 2.76E-11 |
| peroxiredoxin 5 | Prdx5 | 0.62 | 2.01E-40 |
| peroxiredoxin 2 | Prdx2 | 0.64 | 1.06E-41 |
|  | | | |

Genes with a p-value > 0.05 are given in italics. P-value was calculated using differential gene expression analysis (see Supporting Information Methods) by comparing 19 months old WT and 3 months old WT.

**Supplementary Table S3.** Gene enrichment analysis comparing 19 months old Mrps5^V338Y/V338Y^ mutant and Mrps5^WT/WT^ mice. Terms and significance for selected downregulated transcripts in mutants, adjusted p-values are shown.

| **Pathway / GO term / Process Network** | **p-Value corrected** |
| --- | --- |
| Cell adhesion. Cell-matrix interactions (PN) | 1.72E-25 |
| Extracellular matrix organization (GO) | 1.26E-17 |
| ECM-receptor interaction (KEGG) | 1.69E-12 |
| Focal Adhesion WP85 (Wiki) | 2.05E-08 |
| Cell adhesion molecules (KEGG) | 1.92E-06 |
| Complement and coagulation cascades (KEGG) | 2.51E-08 |
| Inflammatory Response Pathway WP458 (Wiki) | 1.75E-04 |
| Neutrophil mediated immunity (GO) | 1.47E-03 |

**Supplementary Table S4:** Primers used for real-time quantitative PCR of target and house-keeping genes.

| **Gene symbol** | **Gene name** | **Primers 5’-3’** |
| --- | --- | --- |
| *Cox11* | Cytochrome c oxidase (OXPHOS complex I) assembly protein 11 | AAG ACG GTG CTC ACC TAC G |
|  |  | GGC ATG AAC GTC AGC ATT GA |
| *Atp5s* | ATP synthase, mitochondrial F0 complex (OXPHOS complex V), subunit S | CAC CAG AAG TGG CTA CAC GA |
|  |  | TCT GTG ACA TTC CCA CAG GC |
| *Mpc1* | Mitochondrial pyruvate carrier 1 | TCA GTG GGC GGA TGA CTT TC |
|  |  | GGC TGG AGC ACT GTC TCT TT |
| *Prdx5* | Peroxiredoxin 5 | TGA ACT TGG CAG AGC TGT TCA |
|  |  | ATA AGT CTG TCG CCT TCC CAA A |
| *Hmbs** | Hydroxymethylbilane synthase | CTG GTC GTT CAC TCC CTG AA |
|  |  | TCT TGA ATT CCA GGT GGG GG |
| *Ywhaz** | Tyrosine 3-monooxygenase activation protein,  zeta polypeptide | GCT TCG CAA CCA GAA AGC AA |
|  |  | GAG AAG TTG AGG GCC AGA CC |
| *Actb** | Beta-actin | CCT CCC TGG AGA AGA GCT ATG |
|  |  | TTA CGG ATG TCA ACG TCA CAC |

* housekeeping genes used as an internal reference
